# Intravenous infusion of mesenchymal stem cells increased axonal signal intensity in the rubrospinal tract in spinal cord injury

**DOI:** 10.1186/s13041-025-01210-0

**Published:** 2025-04-16

**Authors:** Ryosuke Hirota, Masanori Sasaki, Atsushi Teramoto, Toshihiko Yamashita, Jeffery D. Kocsis, Osamu Honmou

**Affiliations:** 1 Department of Neural Regenerative Medicine, Institute of Regenerative Medicine, Sapporo Medical University School of Medicine, S1W17, Chuo-ku, Sapporo, 060-8556 Hokkaido Japan; 2 Department of Orthopaedic Surgery, Sapporo Medical University School of Medicine, Sapporo, 060-8556 Japan; 3 Department of Neurology, Yale University School of Medicine Neurology, PO BOX 208018, New Haven, Connecticut, 06510 USA; 4 Department of Neuroscience, Yale University School of Medicine Neurology, PO BOX 208018, New Haven, Connecticut, 06510 USA; 5 Center for Neuroscience and Regeneration Research, VA Connecticut Healthcare System, West Haven, Connecticut, 06516 USA

**Keywords:** Mesenchymal stem cell, Spinal cord injury, Axon tracing

## Abstract

Limited spontaneous recovery occurs after spinal cord injury (SCI). However, current knowledge indicates that multiple forms of axon growth in spared axons can lead to circuit reorganization. Intravenous infusion of mesenchymal stem cells (MSCs) provides functional improvements after SCI with an increased axonal network. In this study, we examined how intravenous infusion of MSCs facilitates axonal connections in the rubrospinal tract (RST), one of the significant descending tracts, using AAV neuronal tracing techniques. Our finding demonstrated that infused MSCs significantly enhanced axonal signal intensity in the RST, not only around the injury site but also in the rostral and caudal regions, suggesting that neural circuit reorganization is facilitated.

**Supplementary Information:**

The online version contains supplementary material available at 10.1186/s13041-025-01210-0.

Traumatic insults of the spinal cord result in persistent neurological deficits below the level of the lesion. Intravenous infusion of mesenchymal stem cells (MSCs) has shown therapeutic efficacy for spinal cord injury (SCI) via multiple and orchestrated mechanisms, including axonal regeneration, affecting injured spinal tissue [1–4]. Recently, we demonstrated an enhanced axonal network by promoting the axonal signal intensity of pre-existing fine caliber axons among the corticospinal tracts (CSTs) after intravenous infusion of MSCs in a rat model of contused SCI. MSC infusion highlighted this phenomenon of enhanced CST networks when imaged with adeno-associated viral (AAV) vectors, whereas this effect was not evident with vehicle infusion [4].

The rubrospinal tract (RST) is another major descending pathway from the brain to the spinal cord. In rats, the RST originates in the red nucleus of the midbrain and predominantly crosses to the contralateral side at the ventral tegmental decussation. Following this decussation, the RST descends through the lateral-most aspect of the dorsolateral funiculus. This study examined whether intravenous infusion of MSCs promotes areal distribution of the axonal signals in the RST as compared to vehicle infusion in SCI rats.

In the current study, intravenous infusion of MSCs was performed one day after SCI contusion induction. We injected 1.0 × 10^6^ cells in 1.0 mL of fresh DMEM or vehicle (1.0 mL fresh DMEM alone). A SCI was induced at the T9–10 levels using an Infinite Horizons impactor (Precision Systems and Instrumentation, LLC, Lexington, KY, USA) (150-kdyn). As previously reported [1, 3, 4], we confirmed functional improvements following MSC infusion in this model. We used age-matched, untreated rats without SCI induction, vehicle, or MSC infusion as a control group. As an AAV anterograde tracer, tdTomato-encoding AAVs with a CAG promoter (AAV-8-CAG-tdTomato) were used (Vector Biolabs, Malvern, PA, USA). Fourteen days after SCI induction (13 days after MSC or vehicle infusion), SCI and control rats were anesthetized with intraperitoneal injection of ketamine (75 mg/kg) and xylazine (10 mg/kg) and placed in a stereotaxic frame. We injected AAV anterograde tracers into the left red nucleus to visualize the right spinal RST axons (*n* = 5/intact group; *n* = 5/vehicle group; *n* = 5/MSC group). A single injection was made into the red nucleus (AAVs; 4.0 × 10^10^ genome copy/µL, 210 nL) at the following coordinates: 1.0 mm lateral, 8.0 mm depth, and 6.0 mm posterior to the bregma, using a nanoliter-injector (World Precision Instrument Inc., Sarasota, FL, USA) attached to a pulled glass pipette. The needle was left in place for 3 min after injection. The method used in this study allowed us to perform precise micro-delivery of viral vectors to specific brain regions. Histological analysis was performed eight weeks after SCI induction (six weeks after AAV injection). The intact group was perfused at the same time point as the vehicle and MSC groups, six weeks after AAV injection. The rats were perfused transcardially with cold phosphate-buffered saline (PBS), followed by 4% paraformaldehyde, while under deep anesthesia with an intraperitoneal injection of ketamine (75 mg/kg) and xylazine (10 mg/kg). The spinal cords were then dissected and stored at − 80 °C until further use. Sections were cut to a thickness of 50 μm using a cryostat (Sakura Seiki Co, Tokyo, Japan) and washed three times with PBS containing 0.1% Tween 20 (PBS-T). Sections at the C4-5, Th7-8, and L1-2 levels were examined (*n* = 5/group) using a confocal microscope (Zeiss LSM780 ELYRA S.1 system; Carl Zeiss, Jena, Germany) and tdTomato^+^ fluorescence was assessed. The detailed methods used in this study are provided in the supplementary material. We provide a schema of the horizontal spinal cord, displaying the longitudinal levels of coronal sections (green lines) at the C5, Th7, and L2 levels (Fig. 1A). The axonal distribution of RST, visualized using AAV-8-CAG-tdTomato, was located in the lateral funiculus (LF) (Fig. 1B) [5]. In intact animals, tdTomato^+^ RST axons were consistently located in the LF (Fig. 1C1, 1C4, and 1C7) throughout the spinal cord. In vehicle-treated SCI animals, tdTomato^+^ RST axons were more prominently observed rostral to the lesions (Fig. 1C2, 1C5) compared to intact animals. This axonal prominence rostral to the lesions was more evident in MSC-infused animals compared to intact and vehicle-treated groups (Fig. 1C3, 1C6). Although a few dTomato^+^ RST signals were observed caudal to the lesion in the vehicle group (Fig. 1C8), tdTomato^+^ RST signals caudal to the injury in the MSC-infused group appeared at the expected RST position within the LF (Fig. 1C9).

**Fig. 1:**
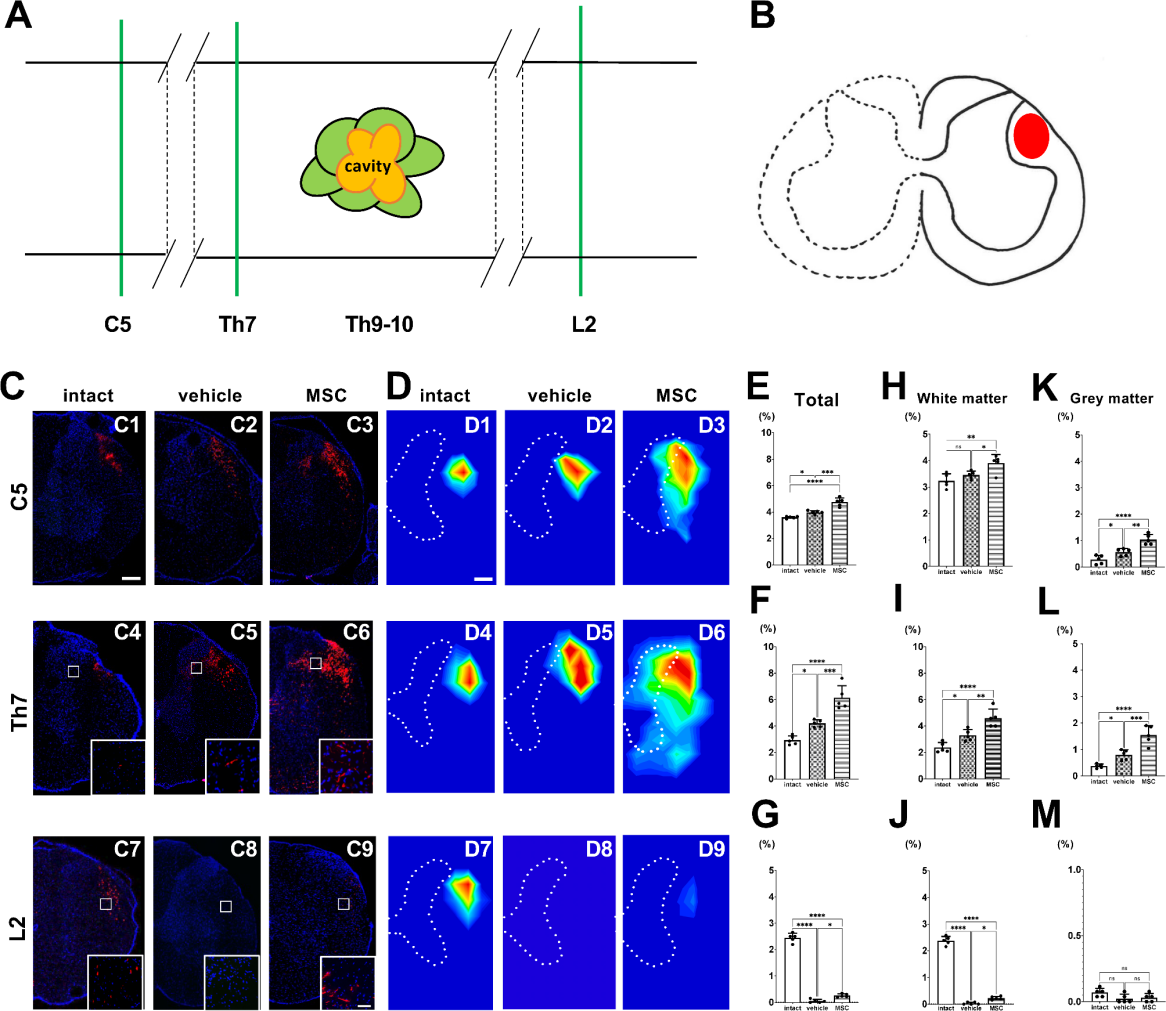
(**A**) Schema of the horizontal spinal cord with cavitation showing areas of confocal analysis rostral (C5 and Th7) and caudal to the lesion (L2). (**B**) Schema of a coronal section of the spinal cord showing the RST visualized by AAV-8-CAG-tdTomato (red) injection. Confocal images of the spinal cord displaying the RST with AAV-8-CAG-tdTomato (red) at cervical (intact, C1; vehicle, C2; MSC, C3), thoracic spinal cord above the injury (intact, C4; vehicle, C5; MSC, C6), and lumbar spinal cord (intact, C7; vehicle, C8; MSC, C9), counterstained with 4’,6-diamidino-2-phenylindole (DAPI, blue). Heatmaps showing axon distribution at the cervical spinal cord (intact, D1; vehicle, D2; MSC, D3), thoracic spinal cord (intact, D4; vehicle, D5; MSC, D6), and lumbar spinal cord (intact, D7; vehicle, D8; MSC, D9). The dashed lines indicate the boundaries of white and gray matter. Quantitative analysis at the (1E for C5, 1 F for Th7, 1G for L2), white matter (1 H for C5, 1I for Th7, 1 J for L2), and gray matter (1 K for C5, 1 L for Th7, 1 M for L2) levels. The y-axis indicates % axonal area (1E-1 M). Data were assessed for normality using the Shapiro–Wilk test. The normally distributed data were analyzed by one-way analysis of variance, and the Tukey–Kramer test was further used to compare post-hoc comparisons. Scale bars: 500 μm (**C, D**), 50 μm (insets). *p < 0.05, **p < 0.01, ***p < 0.001, ****p < 0.0001. (*n* = 5 animals/group), MSC: mesenchymal stem cell

A heatmap generated from the tdTomato^+^ RST signal distribution at C5 (Fig. 1D1-1D3), Th7 (Fig. 1D4-1D6), and L2 (Fig. 1D7–1D9) levels revealed the topographic distribution of RST axons in experimental groups. Quantitative analysis of the areal distribution of the axonal signals in the spinal cord was performed on the side contralateral to the AVV injection in the red nucleus (Fig. 1E for C5, 1F for Th7, 1G for L2), white matter (Fig. 1H for C5, 1I for Th7, 1J for L2), and gray matter (Fig. 1K for C5, 1 L for Th7, 1M for L2). Rostral to the lesions at C5 (Fig. 1E) and Th7 (Fig. 1F), the MSC group showed the highest signal distribution, followed by the vehicle group and then the intact group. Caudal to the lesion at the L2 level (Fig. 1G), the signals in the MSC group were statistically higher than those in the vehicle group. In the white matter (Fig. 1H and I, and 1J) and grey matter (Fig. 1K L, and 1M), the quantitative analyses revealed patterns of statistical significance consistent with those observed in the total spinal cord area at each segmental level, while, there were no statistically significant differences in white matter at the C5 level between the intact and vehicle groups (Fig. 1H), and in gray matter at the L2 level among the groups (Fig. 1M). Of particular interest, rostral to the lesion in the spinal cord, the vehicle group exhibited a greater areal distribution of the axonal signals on the side contralateral to the AVV injection in the red nucleus compared to the intact group, which was further enhanced by the MSC group (Fig. 1E and F). These findings indicate that, as a response to SCI, axonal plasticity is enhanced even in cervical regions distant from the lesion site and that MSC administration further amplifies this phenomenon. Additionally, at caudal levels, the areal distribution of the axonal signals in the MSC group was significantly greater than in the vehicle group in the white matter (Fig. 1J), not in gray matter (Fig. 1M).

Although the exact mechanisms remain to be elucidated, the probability of the number of converging axons beyond the lesion is increased. Therefore, the regeneration of the RST in the current study might also contribute to functional improvements following intravenous infusion of MSCs in contused SCI [6]. In addition, our findings in the previous paper provided another possibility that axonal enhancement of small caliber axons is more likely than frank regeneration or sprouting [4]. It is unlikely that the injured axons traveled through the hostile environment and precisely rejoined the dorsal cortical spinal tract to the lesion. Injured CST components formed an enhanced axonal network parallel to minor CST components, converging back to the cross-sectional position of the dorsal CST (dCST) caudal to the lesion core. Only MSC infusion highlighted this phenomenon of these enhanced axons when imaged with adeno-associated viral (AAV) vectors, whereas this effect was not evident with vehicle infusion. The pre-existing fine caliber axons, which are not readily detectable by conventional tracing methods, increase in diameter and contribute to improved conduction to motor neurons below the SCI site [4]. It is conceivable that this novel mechanism also operates in RST. Finally, it should be noted that numerous unrecognized axonal networks may still exist within the spinal cord, and conventionally-undetectable fine caliber axons increase their diameter and become emergent after MSC infusion, contributing to functional improvement [4]. In particular, the AAV anterograde tracer provides more precise analysis by improving spatial resolution compared to conventional imaging techniques, allowing us to easily detect the newly-enhanced axonal networks following MSC injection. The technique used in this study has the potential to be widely adopted for refined analysis of specific axons because this non-transsynaptic axonal tracer has the advantage of tracing only the axon, avoiding the projection to the interneuron in the spinal cord. Thus, this technique would allow us to separately elucidate regenerative patterns in a variety of neural tracts within the spinal cord after intravenous infusion of MSCs. We point out that there are limitations in the interpretation of the red nucleus AAV labeling results. First, although all AAV injection procedures were performed using identical stereotaxic coordinates and injection volumes to target the red nucleus with precision, we cannot rule out the possibility of some ectopic labeling of neurons near the red nucleus.　Second, this study has a detection limit because axons were directly observed using tdTomato^+^ signals under confocal microscopy. Specifically, only axons above a signal detectable diameter could be detected, while finer-caliber axons may not have been visualized. Third, we cannot rule out the possibility that infused MSCs affected AAV viral transduction. However, since AAV was administered two weeks after MSC infusion, any potential influence of MSCs should be minimal.

## Electronic supplementary material

Below is the link to the electronic supplementary material.

Supplementary Material 1

## Data Availability

No datasets were generated or analysed during the current study.
