# Supplementary material for: Intravenous infusion of mesenchymal stem cells increased axonal signal intensity in the rubrospinal tract in spinal cord injury
Source: Mol Brain. 2025 Apr 16;18:35. doi: 10.1186/s13041-025-01210-0 (PMC12004759; doi:10.1186/s13041-025-01210-0)
Supplement: Supplementary file 1 — Supplementary Material 1 [file 13041_2025_1210_MOESM1_ESM.docx]

**Online Supplementary Material for**

**Intravenous infusion of MSCs increased axonal signal intensity in the rubrospinal tract in spinal cord injury**

**Authors:**

Ryosuke Hirota ^1, 2^, Masanori Sasaki ^1, 3^, Atsushi Teramoto^2^, Toshihiko Yamashita ^2^, Jeffery D. Kocsis ^3, 4, 5^, Osamu Honmou ^1, 3^

**Affiliations:**

1. Department of Neural Regenerative Medicine, Institute of Regenerative Medicine, Sapporo Medical University School of Medicine, Sapporo, 060-8556, Japan

2. Department of Orthopaedic Surgery, Sapporo Medical University School of Medicine, Sapporo, 060-8556, Japan

3. Department of Neurology, Yale University School of Medicine

Neurology, PO BOX 208018, New Haven, Connecticut, 06510, USA

4. Department of Neuroscience, Yale University School of Medicine

Neurology, PO BOX 208018, New Haven, Connecticut, 06510, USA

5. Center for Neuroscience and Regeneration Research, VA Connecticut Healthcare System, West Haven, Connecticut, 06516, USA

**Methods**

**Animals**

All experiments were conducted in accordance with the institutional guidelines of Sapporo Medical University. The use of animals in this study was approved by the Animal Care and Use Committee and the Committee for Security of Recombinant DNA Experiments of Sapporo Medical University.

**Preparation of mesenchymal stem cells from rat bone marrow**

MSC preparation and culture were conducted based on our previous studies (1). Briefly, bone marrow obtained from femoral bones in adult (6–8 weeks old) Sprague-Dawley (SD) rats was diluted to 15 mL with Dulbecco's modified Eagle's medium (DMEM) (Sigma, St. Louis, MO, USA) supplemented with 10% heat-inactivated fetal bovine serum (Thermo Fisher Scientific Inc., Waltham, MA, USA), 2 mM l-glutamine (Sigma), 100 U/ml penicillin, and 0.1 mg/mL streptomycin (Thermo Fisher Scientific Inc.) and incubated for 3 days at 37 °C in a humidified atmosphere containing 5% CO_2_. When cultures almost reached confluence, the adherent cells were detached with a trypsin-ethylenediaminetetraacetic acid solution (Sigma) and subcultured at 1 × 10^4^ cells/ml of medium. After three passages, the MSCs were used in the present study. A previous phenotypic analysis of the surface antigens revealed cluster of differentiation (CD) 45^-^, CD73^+^, CD90^+^, and CD106^-^ on MSCs.

**SCI model**

Contusive SCI was performed as described previously (1). Briefly, adult (7-week-old) male SD rats (250–300 g) were anesthetized with an intraperitoneal injection of ketamine (90 mg/kg, intraperitoneal) and xylazine (4 mg/kg, intraperitoneal). After skin incision, the T9 vertebra was stabilized, a laminectomy was performed at the T9–10 level of the spinal cord, and a 150-kdyn contusion was delivered to the spinal cord using an Infinite Horizons impactor (Precision Systems and Instrumentation, LLC, Lexington, KY, USA). Appropriate postoperative care was provided for all animals, including twice-daily manual bladder expression for up to 14 days. The rats were housed in an atmosphere of 50% humidity at a temperature of 24 ± 2°C.

**Experimental protocol**

Only rats with Basso, Beattie Bresnahan (BBB) open field locomotor scores (2) of zero one day after SCI induction were included in this study. These SCI rats were randomized and received a single intravenous infusion of MSCs at 1.0 × 10^6^ cells in 1.0 mL of fresh DMEM or vehicle (1.0 mL fresh DMEM alone) via the femoral vein one day after SCI induction. All rats were injected daily with cyclosporine A (10 mg/kg, IP). Age-matched intact rats were used as intact controls. On day 14 after SCI induction, the AAV virus was injected into SCI animals and age-matched intact animals (see Neuroanatomical tracing). Histological analysis was performed eight weeks after SCI induction (6 weeks after AAV injection).

**Histological analysis**

The rats were perfused transcardially with cold phosphate-buffered saline (PBS), followed by 4% paraformaldehyde, while under deep anesthesia with an intraperitoneal injection of ketamine (75 mg/kg) and xylazine (10 mg/kg). The spinal cords were then dissected and stored at −80 °C until further use. Sections were cut to a thickness of 50 μm using a cryostat (Sakura Seiki Co, Tokyo, Japan) and washed three times with PBS containing 0.1% Tween 20 (PBS-T). Sections at the C4-5, Th7-8, and L1-2 levels were examined (n = 5/group) using a confocal microscope (Zeiss LSM780 ELYRA S.1 system; Carl Zeiss, Jena, Germany) and tdTomato^+^ fluorescence was assessed. Fluorescence signals were excited using a laser at a wavelength of 561 nm and detected within the range of 550–600 nm. To analyze the three-dimensional structure of the spinal cord, Z-stack imaging was performed to acquire images at different depths. Images were acquired using a 20× objective lens with a 1 μm Z-axis interval, resulting in a total of 20 slices spanning a range of 0–20 μm. The Z-stack images were processed using ImageJ software bundled with Java 1.8.0_172 (National Institutes of Health, Bethesda, MD, USA; <http://rsv.info.nih.gov/ij/>) to generate 2D projections (Fig. 1C).

The TdTomato fluorescence-positive axonal area in a confocal image was quantified using ImageJ. To ensure consistency, spinal cord sections were oriented based on the dorsal-ventral and medial-lateral axes before analysis. A central canal was used as a reference point for both signal intensity and the anatomical landmark of the spinal cord. 8×12 region of interest (ROI) were placed on the area of 4000 μm × 6000 μm, including the hemispinal cord. One spinal section per spinal level (C4-5, Th7-8, and L1-2) was analyzed. Individual values from each ROI of five animals were averaged. The boundaries between white and gray matter were determined based on confocal images from each sample. Fig.1D shows that the averaged individual value of ROIs was assigned to 20 divisions ordered from high to low numbers, with the squares in the highest division represented in red and the lowest in blue by GraphR as a heatmap. The value of tdTomato fluorescence-positive axonal area in confocal images was quantitatively analyzed and demonstrated in the hemispinal cord (Fig. 1E-1G), white matter (Fig. 1H-1J), and grey matter (Fig. 1K-1M).

**Statistics**

All statistical analyses were performed using the Statistical Package for the Social Sciences 21 for Macintosh (IBM, Inc., IL, USA). Data were assessed for normality using the Shapiro–Wilk test. The normally distributed data were analyzed by one-way analysis of variance, and the Tukey–Kramer test was further used to compare post-hoc comparisons. Data are expressed as mean ± standard error of the mean. Differences were considered statistically significant at p < 0.05.

1. Hirota R, Sasaki M, Kataoka-Sasaki Y, Oshigiri T, Kurihara K, Fukushi R, et al. Enhanced Network in Corticospinal Tracts after Infused Mesenchymal Stem Cells in Spinal Cord Injury. J Neurotrauma. 2022;39(23-24):1665-77.

2. Basso DM, Beattie MS, Bresnahan JC. A sensitive and reliable locomotor rating scale for open field testing in rats. J Neurotrauma. 1995;12(1):1-21.
